# Prevalence, Risk Factors, and Management of Irritable Bowel Syndrome in Saudi Arabia: A Systematic Review

**DOI:** 10.7759/cureus.47440

**Published:** 2023-10-21

**Authors:** Eyad A Makkawy, Israa E Abdulaal, Farah R Kalaji, Mohammed Makkawi, Nasser Alsindi

**Affiliations:** 1 Internal Medicine/Gastroenterology Department, Prince Mohammed Bin Abdulaziz Hospital, Riyadh, SAU; 2 Health Sciences Department, Princess Nourah Bint Abdulrahman University, Riyadh, SAU; 3 Emergency Department, King Fahad General Hospital, Medina, SAU; 4 Public Health Department, Medina Health Cluster, Medina, SAU

**Keywords:** ibs, kingdom of saudi arabia (ksa), ibs (irritable bowel syndrome), inflammatory bowel disease, ibd

## Abstract

The prevalence and associated risk factors of irritable bowel syndrome (IBS) have been a significant area of focus in several studies conducted in Saudi Arabia. These studies have looked at varied populations, including school teachers, university students, and the general populace. The reported prevalence rates for IBS vary substantially across studies, ranging from 7.9% to an astounding 49.3%. The average prevalence noted across these studies is about 24%. The aim of this review is to collate, compare, and analyze data from these studies, hoping to shed light on the key risk factors and demographic trends associated with IBS in Saudi Arabia. This review encompasses data from 20 studies, aggregating information from 17,018 participants. The research methodologies adopted by each of these studies have been analyzed, especially focusing on their sample sizes, which vary significantly. Furthermore, the review incorporates details on the socio-demographic attributes of the participants, including age specifics, gender representation, and geographical distribution within Saudi Arabia. The results demonstrate a wide variability in IBS prevalence among different groups. The overall prevalence of IBS in Saudi Arabia based on the provided data is approximately 24%. Gender-based breakdown in some studies indicated varying prevalence among males and females, which indicated that females are more prone to the disease. The same for certain age groups, specifically between 51 and 60 years, which showed slightly higher rates. Factors such as educational discipline, living conditions, mental health, dietary habits, family history of IBS, and certain comorbidities such as diabetes mellitus were found to influence the occurrence of IBS in different cohorts. Moreover, lifestyle factors such as low water intake, lack of dietary fiber, stress, and even caffeine intake were associated with IBS. Socioeconomic aspects, including family income levels and academic performance, were also hinted to have a potential link with IBS prevalence. In light of the presented data, it is evident that IBS prevalence in Saudi Arabia is influenced by a multitude of factors, ranging from genetic and dietary to psychological and socioeconomic. The substantial variations in prevalence across different cohorts suggest the need for a more nuanced understanding of this condition, specifically tailored to the unique demographics and cultural contexts of Saudi Arabia. Early diagnosis and tailored interventions, considering these multifaceted determinants, are crucial for the effective management of IBS in the region.

## Introduction and background

Irritable bowel syndrome (IBS) stands as one of the most prevalent gastrointestinal disorders affecting the global population. Characterized by a myriad of symptoms, including abdominal pain, bloating, and varying bowel habits, this condition has garnered significant attention from the medical community due to its profound impact on patients' daily lives [1]. Its multifaceted etiology encompasses both environmental triggers and inherent genetic susceptibilities [2], which can differ across geographies and cultures.

Despite its widespread occurrence, the management and understanding of IBS remain complex. The syndrome does not merely perturb the physical wellbeing of the affected individuals but also exacts a psychological toll. This, coupled with the chronic trajectory of the condition, presents both direct and indirect challenges to healthcare systems worldwide [3]. Direct challenges stem from the medical interventions and diagnostics required for managing IBS, while indirect challenges relate to reduced work productivity, increased absenteeism, and the overall decreased quality of life of the patients [3].

From a global standpoint, the prevalence and manifestation of IBS display variability based on numerous factors, including the region, the diagnostic criteria employed, and the methodologies underpinning the respective studies [4]. Within this global tapestry, Saudi Arabia emerges as a particularly interesting subject of study. The rapid socio-cultural and economic transformations witnessed by the country over the past few decades may have implications for lifestyle and dietary habits, which in turn could influence IBS prevalence and its associated risk factors. However, systematic data delving into the intricacies of IBS prevalence, its risk factors, and the contemporary management paradigms in Saudi Arabia remain sparse.

This systematic review, therefore, embarks on a mission to collate and analyze the existing body of research centered on IBS in Saudi Arabia. By providing a panoramic view of the current landscape of IBS within this specific geographical context, the review hopes to pave the way for more targeted research, refined diagnostic strategies, and patient-centric management interventions.

IBS has been increasingly recognized as a significant global health concern, with its prevalence and impact varying across continents and countries. Understanding the global epidemiology of IBS is crucial not only for healthcare policymakers and practitioners but also for researchers aiming to decipher the environmental and genetic determinants of this multifaceted condition.

Globally, the prevalence of IBS is estimated to be around 11.2% [5]. However, this value displays considerable variation across different regions. While some Western countries report prevalence rates as high as 20%, other regions, such as South Asia and the Middle East, report rates between 7% and 10% [5].

In North America, particularly in the United States, the prevalence of IBS is estimated to be around 10-15% [6]. European countries show a similar prevalence range, with the United Kingdom highlighting rates of approximately 10-20% [7]. Asian countries present a diverse epidemiological picture. A study conducted in South Korea reported a prevalence rate of around 8.0% [8], whereas in India, the prevalence is estimated to be approximately 4.2% [9,10].

The heterogeneity in global IBS prevalence figures can also be attributed to the use of different diagnostic criteria across studies. Rome III and Rome IV are the most commonly employed criteria; however, differences in their application and interpretation can lead to variations in reported prevalence [11].

The management of IBS is complex, requiring an individualized and multifaceted approach. Current recommendations encompass both non-pharmacological and pharmacological strategies tailored to symptom severity and patient preference. Here are the primary interventions that have shown efficacy in the clinical management of IBS [12-15].

## Review

Methodology

The Preferred Reporting Items for Systematic Reviews and Meta-Analyses (PRISMA) guidelines were followed for this systematic review.

Study design and duration

This was a systematic review conducted in August 2023.

Search Strategy

To retrieve relevant research, a thorough search was conducted across five major databases, including Google Scholar, PubMed, Web of Science, Science Direct, and EBSCO. We only searched in English and took into account each database's unique criteria. The following keywords were converted into PubMed Mesh terms and used to find studies that were related: “IBD,” " Inflammatory Bowel Disease," "Prevalance," "Managment," "Saudi Arabia,” and "Risk Factors." The Boolean operators "OR" and "AND" matched the required keywords. Among the search results were publications in full English language, freely available articles, and human trials.

Selection Criteria

We considered the following criteria for inclusion in this review: any study designs that investigate the prevalence of IBS in KSA, no age limit restriction, and free accessible articles.

For the exclusion criteria, we excluded studies before June 2016, studies outside Saudi Arabia, case reports, letters to the editors, replies to conflicts, and studies in non-English language.

Data Extraction

Duplicates in the search strategy output were found using Rayyan (QCRI; Rayyan Systems Inc., Cambridge, MA) [16]. To determine the titles’ and abstract relevance, the researchers used a set of inclusion/exclusion criteria to filter the combined search results. The reviewers carefully read each paper that matches the requirements for inclusion. The authors provided other methods of resolving disputes with some thought. The authors extracted data about the study titles, authors, study year, country, participants, gender, diagnostic tool, main outcomes, and conclusion.

Strategy for Data Synthesis

Summary tables were created using information from pertinent research to give a qualitative overview of the results and study components. Following data extraction for the systematic review, the most effective strategy for utilizing data from the included study articles was selected.

Risk of Bias Assessment

Using the ROBINS-I risk of bias assessment approach for non-randomized trials of therapies, the included studies' quality was assessed [17]. The seven themes that were assessed were confounding, participant selection for the study, classification of interventions, deviations from intended interventions, missing data, assessment of outcomes, and choosing of the reported result.

Results

Search Results

**Figure 1:**
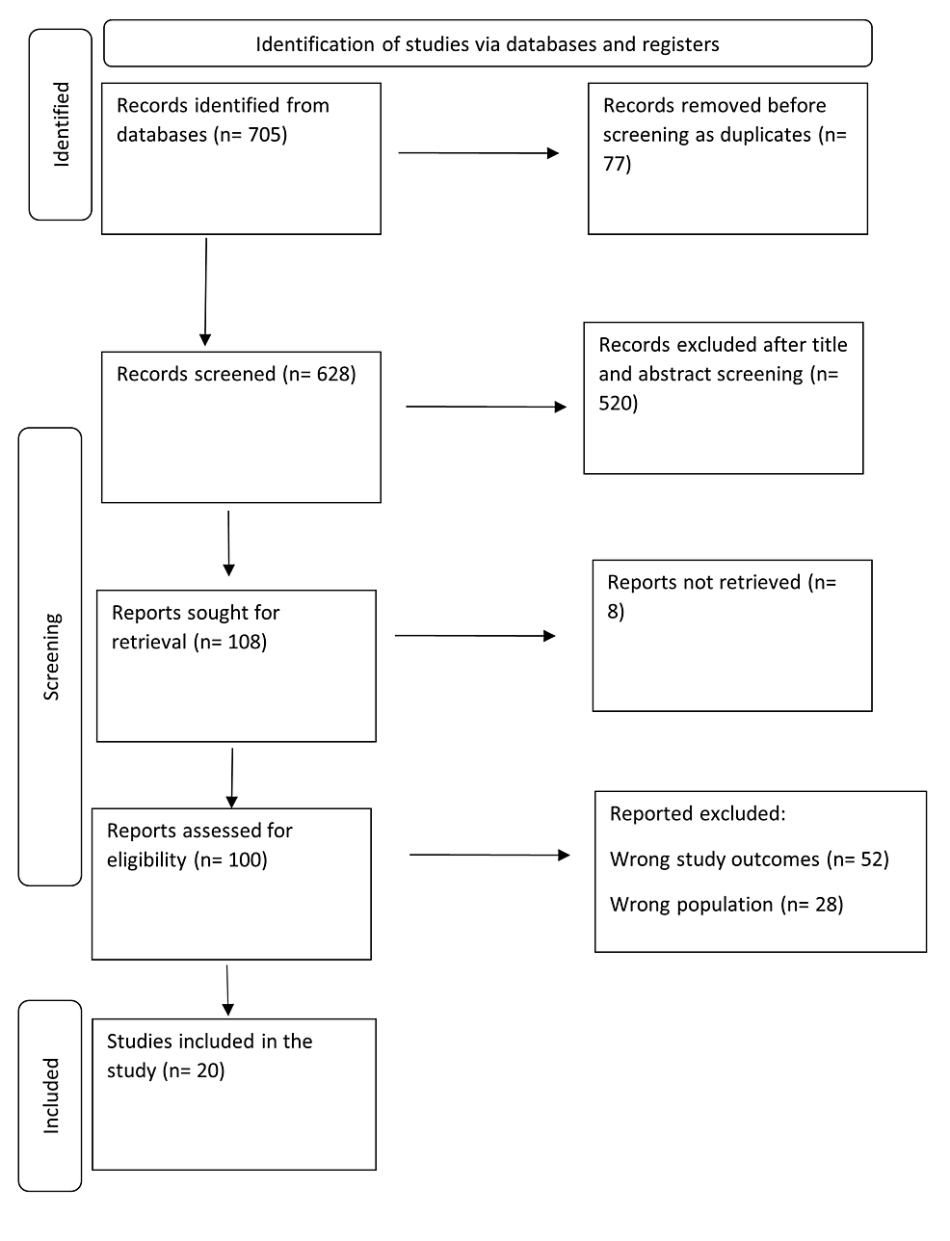
PRISMA flowchart summarizing the study selection process.

The systematic search yielded 705 study papers, of which 77 duplicates were removed. On 628 studies, title and abstract screening was performed, and 520 studies were eliminated. Only eight items were not recovered out of 108 that were searched for retrieval. Finally, 100 papers were examined for full-text evaluation; 52 were removed due to incorrect research outcomes, as well as 28 for the incorrect population category. This systematic review comprised 20 study papers. A synopsis of the research selection procedure is provided in Figure 1.

Characteristics of the Included Studies

Table 1 delves into the sociodemographic characteristics and research methods of the studies under review. Altogether, our compilation integrates 20 studies, cumulating a total of 17,018 participants [18-37]. The sample sizes within these studies vary, from the smallest at 115 [37] to the largest being 6,300 participants [27]. The male representation across these studies displays substantial variation, from the absence of male participants in the study from Arar [36] to a full male cohort in the studies from Najran [24] and Majmaah [37]. When averaged across all studies, the male representation equates to roughly 63%.

**Table 1:** Sociodemographic characteristics of the included participants.

Study	Study design	Location	Participants	Age Range and (age average)	Males (%)
AlKhalifah et al. 2016 [18]	Cross-sectional	Qassim region	300	-	-
Hakami et al. 2019 [19]	Cross-sectional	Jazan University	825	(21.69)	46%
Alharbi et al. 2022 [20]	Cross-sectional	Makkah	921	(30.32)	40%
Amin et al. 2021 [21]	Cross-sectional	Riyadh	1,319	(35)	53.5%.
Alqahtani et al. 2019 [22]	Cross-sectional	All KSA	1,680	(32.2 ± 12.3 years)	36%
Alharbi et al. 2019 [23]	Cross-sectional	Hail	930	(33)	42%
Saad Alshahrani (2020) [24]	Cross-sectional	Najran	400	(22.5)	100%
Alnasser et al. 2023 [25]	Snowball sampling technique, survey	Qatif	279	(38.38)	43.7%
Ibrahim et al. [26]	Cross-sectional	Jeddah	525	(21.38 ± 2.09)	31.4%
Basharat et al. 2022 [27]	Cross-sectional	Abha, Aseer	6300	-	66.7%
Sachithananthan (2018) [28]	Cross-sectional	Abha	150	(30)	46.7%
AlAmeel et al. 2019 [29]	Cross-sectional	KSA	594	(41)	70.5%
Ahmed et al. 2020 [30]	Cross-sectional	Riyadh	472	18-26	60.9%
Aljammaz et al. 2020 [31]	Cross-sectional	Central Region	426	14-65	54%
Hafiz et al. 2023 [32]	Cross-sectional	Makkah	936	36.19	45.5%
Taha et al. (2019) [33]	Cross-sectional	Al-Madinah	205	34.5	50.2%
Bashir Fadl et al. 2022 [34]	Cross-sectional	KSA	300	-	36.3%
Hasosah et al. 2017 [35]	Cross-sectional	Jeddah	179	20-26	83.8%
Alshammari et al. 2016 [36]	Cross-sectional	Arar	207	36 ± 7.5	0%
Aldawsari et al. 2017 [37]	Cross-sectional	Majmaah	115	-	100%

From a geographical standpoint, the studies spanned multiple regions within Saudi Arabia. These are Qassim [18], Jazan [19], Makkah (featured in two studies) [20,32], Riyadh (represented in two papers) [21,30], throughout KSA [22,29,34], Hail [23], Najran [24], Qatif [25], Jeddah (detailed in two instances) [26,35], Abha (occurring in two studies) [27,28], Central Region [31], Al-Madinah [33], Arar [36], and finally Majmaah [37].

The predominant research design among the studies is the cross-sectional approach [18-23,26-28,29-33,35-37]. Only one study stood out by employing the snowball sampling technique [25]. Age parameters are diversified across the studies. Some provide specific average ages such as 36.19 years [32], while others offer them with standard deviations (e.g., 36 ± 7.5 years) [36]. Several studies proffer age ranges, exemplified by 14-65 years [31]. Unfortunately, a handful did not specify age details [18,27,34,37]. Using the data from studies that rendered distinct age averages, the consolidated mean age is approximately 28.15 years.

Table 2 presents the clinical characteristics of the included studies.

**Table 2:** Clinical characteristics and outcomes of the included studies.

Study	Prevalence	Risk Factors	Management and outcomes
AlKhalifah et al. 2016 [18]	40.7% or 122 of the 300. Only 35.2% had been previously diagnosed with IBS. A breakdown revealed a slightly higher prevalence among males (43.3%) than females (38%). Additionally, IBS prevalence was marginally more common among teachers aged 51-60 years (44.4%, and 31-40 years (42.9%).	Teachers between the ages of 31-40 had the highest representation in the study at 54.7% (164/300). Those aged between 41-50 were 25.3% (76/300), while both age brackets 21-30 and 51-60 had fewer teachers at 17% (51/300) and 3% (9/300) respectively.	Teachers with IBS showed a more pronounced tendency to miss work compared to their peers. Specifically, 46.6% of teachers with IBS were absent from their jobs at least once a month, contrasting with just 25% of teachers without IBS doing the same.
Hakami et al. 2019 [19]	Of the initial 890 participants, 65 were excluded due to incomplete data, resulting in a final sample size of 825 students, a response rate of 92.7%. Among the respondents, the overall prevalence of IBS, determined using the Rome IV Criteria, was 8.8%. IBS prevalence was found to be slightly higher in female students (10.0%) than male students (7.6%). The breakdown by subtype revealed that 31.5% had IBS-C, 28.8% had IBS-M, 21.9% had IBS-D, and 17.8% had IBS-U.	The study unveiled several risk factors associated with IBS: Educational Discipline: IBS prevalence was highest among students of scientific colleges at 16.5%, compared to humanities at 3.4%. Living Conditions: IBS was more prevalent among students living on campus (26.7%) compared to those living in rented apartments (9.4%) or with their families (7.6%). Behaviors and Health Background: There was a statistically significant association of IBS with cigarette smoking, Khat chewing, food intolerance, a history of travel diarrhea, a family history of IBS, and emotional stress in the past 6 months. Mental Health: While no significant difference was found between IBS and anxiety, the prevalence of depression was slightly higher in the IBS group (16.2%) compared to the non-IBS group (13.9%).	The study revealed an 8.8% prevalence of IBS among Jazan University students, with females (10%) slightly more affected than males (7.6%). IBS occurrence was highest in scientific college students, particularly medical students. Living on campus and behavioral factors like smoking, Khat chewing, and food intolerance also increased the risk. Emotional stress in the recent 6 months was significantly linked to IBS. Despite a marginally higher rate of depression among IBS students, there was no notable difference in anxiety levels between IBS and non-IBS groups.
Alharbi et al. 2022 [20]	A total of 921 individuals from Makkah completed the questionnaire. The overall IBS prevalence was 20.19%. There were distinct subtypes for IBS, with IBS-M (mixed subtype) being the most common at 53.8%, followed by IBS-C (constipation subtype) at 22%, IBS-D (diarrhea subtype) at 15%, and IBS-U (unsubtyped) at 8.6%.	Participants above 60 years had the highest IBS prevalence at 25%. Females exhibited a slightly higher prevalence of 21% compared to males at 19%, although this wasn't significant. Past smokers had a 23.1% likelihood of having IBS compared to current smokers at 22.3% and non-smokers at 19.7%. This difference, however, wasn't statistically significant. Those with depression had a 25.6% prevalence of IBS, anxiety at 25.5%, and stress at 28.7%, with stress having a P-value of <0.001.	The association between IBS and psychiatric disorders was further analyzed. Stress was found to have a significant association with IBS, However, anxiety and depression were not found to be significant risk factors for IBS, with P-values of 0.149 and 0.367, respectively.
Amin et al. 2021 [21]	Of the participants, IBS was diagnosed in 104 subjects, constituting a prevalence rate of 7.9%. Among them, 52% had IBS-M (mixed) type. A higher prevalence was observed in women (4.9%) than in men (3.0%). The self-reported prevalence among those who believed they had IBS symptoms was 26%, but only 7.9% of them met the Rome IV criteria. The age group with the highest rate of IBS was the younger participants (59.6%).	There was a significant association between the occurrence of IBS symptoms and several sociodemographic factors. Women had a higher prevalence of IBS than men. IBS was notably linked to low income and unemployment. Age, educational level, and marital status did not show a significant correlation with IBS.	The study examined the dietary preferences of IBS patients. Those with IBS were more inclined to avoid specific foods compared to those without IBS. The most commonly restricted foods were legumes (65.4%), milk (54.8%), and fatty food (46.2%), among others. The study also evaluated knowledge about herbal drinks (mint, ginger, chamomile) used for pain relief among participants. The knowledge level among IBS patients about these herbs was lower, but the difference wasn't statistically significant.
Alqahtani et al. 2019 [22]	IBS was found to be prevalent in 18.2% of the study participants. When looking at IBS subtypes, the most commonly reported subtype was IBS-M, accounting for 42.3%. Other subtypes like IBS-C, IBS-D, and IBS-U made up 27.2%, 21.6%, and 8.8%, respectively.	Certain risk factors exhibited a significant association with IBS. These include smoking habits, gastroesophageal reflux disease (GERD), food allergies, anxiety, psychological stress, a family history of IBS, regular intake of non-steroidal anti-inflammatory drugs (NSAIDs), a history of infection preceding the appearance of IBS symptoms, and residing in the southern part of Saudi Arabia. However, no significant associations were observed between IBS and factors such as ABO blood groups, history of pelvic surgery, the use of oral contraceptive pills, or the use of antibiotics prior to the onset of IBS symptoms. The most frequently observed risk factor among IBS patients in the study was a family history of IBS, with 80% reporting it.	The study concludes by emphasizing the need to raise public awareness regarding IBS in Saudi Arabia and advocates for additional prospective studies on the subject.
Alharbi et al. 2019 [23]	The study found that the prevalence of IBS was 11% among males and 12.5% among females. Meanwhile, the prevalence of IBS symptoms was higher, standing at 30% for males and 36.5% for females. Additionally, prevalence rates varied across different age groups, with the highest prevalence of IBS symptoms seen in the 30-39 age bracket (42%).	The data suggested that there's a significant association between IBS and reduced water intake, with a relative risk (RR) of 1.1800 (95% CI of 1.0146-1.3722) and a p-value of 0.0316. Remarkably, 97.3% of the study population wasn't accustomed to consuming vegetables/fruits. Moreover, while a significant proportion (85.5%) of the participants consumed caffeine, its consumption didn't significantly correlate with IBS. The study also explored other dietary habits such as home food, fast food, and spicy food, but none showed a statistically significant association with IBS.	IBS is commonly observed in Northern Saudi Arabia, affecting both genders but showing a slight rise among females. The increasing occurrence of IBS in the region might be attributed to inadequate consumption of water and a lack of dietary fiber, especially from fruits and vegetables.
Saad Alshahrani et al. (2020) [24]	Out of the 400 male secondary school students, 159 (or 39.8%) displayed symptoms indicative of IBS. The most prevalent type of IBS observed was IBS-M (alternating diarrhea and constipation) at 26.3%. This was followed by IBS-D (diarrhea) at 7.3% and IBS-C (constipation) at 6.3%.	Several risk factors were associated with a higher prevalence of IBS among male secondary school students. Specifically, a positive family history of IBS and the presence of diabetes mellitus were significantly linked to increased IBS prevalence. When examined closely, students with a positive family history of IBS had a prevalence rate of 56.3%, while those without it had a rate of 32%. Additionally, diabetic students showed a remarkably higher IBS prevalence (83.3%) compared to non-diabetic ones (38.4%).	The study concluded by emphasizing the importance of providing health education to secondary school students to equip them with knowledge on how to manage and mitigate IBS symptoms.
Alnasser et al. 2023 [25]	IBS was found to be prevalent in 17.6% of the participants. Among these, the condition was more common in older adults, with a prevalence rate of 31.3%, and more frequent in women at 20.4%.	The study found a statistically significant association of IBS with age and gender. It was more prevalent among older women. Females had an odds ratio (OR) of 2.067, suggesting they were more likely to have IBS than males. Similarly, older-aged adults had an OR of 4.235, indicating a higher likelihood of having IBS.	the study emphasizes the negative impact of IBS on HRQoL, especially among elderly women. Measures to manage and alleviate symptoms of IBS could improve Health-related quality of life (HRQoL) for these patients.
Ibrahim et al. [26]	One-third (33.3%) of the paramedical students met the Rome-III criteria for IBS. The most prevalent subtype of IBS was IBS-Mixed (IBS-M) at 58.9%. Nursing (41.7%) and dentistry (37.8%) students reported the highest IBS prevalence, while those from the nutritional specialty had the lowest. The study also found that only 24.0% of students who met the IBS criteria were previously diagnosed by physicians.	IBS was significantly associated with various factors including the following. Gender: Higher prevalence in females (38.1%) than in males (20.8%). Educational specialty: Particularly high in nursing and dentistry students. Family history of IBS. Presence of chronic medical diseases. Experiencing traveler's diarrhea. Food hypersensitivity. Poor sleep quality: Prevalence of poor sleep quality was 68.6% among all participants. Psychological stress, anxiety, and depression.	Management: The study suggests that screening for IBS and psychological issues, introducing stress management courses, and IBS educational programs would be beneficial for paramedical students given the high prevalence and associated factors found in the study.
Basharat et al. 2022 [27]	23.81% of the respondents (1500) had IBS	When analyzing daily activities and conditions, 71.4% worked 6-8 hours a day, 30.2% were students, and 26.7% were housewives. It was observed that 71.4% resided in cities, while the remaining lived in villages. In terms of health and lifestyle, 61.9% were married, 26.1% had diabetes, 23.2% had hypertension, 28.6% were current smokers, and 47.6% were non-smokers, with the rest having quit smoking.	The primary conclusion drawn was a prevalence rate of 23.81% for IBS in the south-west area of Saudi Arabia. Moreover, IBS was found to be significantly associated with being female, tobacco smoking, and mental health issues.
Sachithananthan (2018) [28]	The prevalence of IBS was found to be higher in women than men, with an overall prevalence of 53.3% among the participants.	64% of the subjects either had a history of colon disease or knew someone who had. Psychological stress or anger led to IBS symptoms in 61.3% of the subjects. Even though 75% of subjects were non-smokers, the remaining 25% still smoked	51.3% of the subjects did not regularly consult with a physician. 30.7% of the subjects did not adhere to or pay attention to doctors' instructions.
AlAmeel et al. 2019 [29]	The overall prevalence of IBS among the surveyed physicians and surgeons in Saudi Arabia was determined to be 16.3%. When broken down by specialty, the prevalence was 16.2% for gastroenterologists, 14.3% for surgeons, and 15.3% for pediatricians. In terms of IBS types, mixed IBS was the most prevalent, affecting 45.4% of those diagnosed, followed by diarrhea-predominant IBS at 32%, constipation-predominant IBS at 20.6%, and unsubtyped IBS at 2.1%.	A binary logistic regression analysis revealed that age, gender, and work hours significantly predicted the likelihood of having IBS. Specifically, younger age increased the odds (OR = 0.931, P < 0.0001), being male decreased the odds (OR = 0.504, P = 0.003), and longer work hours increased the odds (OR = 2.397, P < 0.0001) of IBS presence.	longer working hours, gender, and age play roles in predicting IBS can lead to better support and resources for affected individuals in the medical field.
Ahmed et al. 2020 [30]	Of the 294 medical students whose responses were included in the data analysis, 37 students, or 12.6%, were identified with symptoms consistent with IBS. Subtypes of IBS reported were IBS-M at 48.6%, IBS-D and IBS-C both at 21.7%, and IBS-U at 8%.	A significant association was found between the prevalence of IBS and smoking, with an odds ratio of 3.351 (p=0.032). A higher prevalence of IBS was observed in females, although this was not statistically significant. Also, a higher prevalence of IBS was noted among third- and fifth-year students.	To address the identified risk factor, it was proposed that a smoking rehabilitation program be initiated to help students abstain from the habit, which could potentially reduce the risk of IBS development in the future.
Aljammaz et al. 2020 [31]	out of 426 subjects who participated in the study, 130 had IBS, indicating a prevalence of 30.5%. Furthermore, based on the Hospital Anxiety and Depression Scale (HADS), 59 participants (13.8%) had depression, and 120 participants (28.2%) had anxiety.	The study identified several factors with statistically significant associations with symptomatic IBS. These include gender, anxiety, depression, and low physical activity. However, factors such as smoking; family history of IBS; inadequate sleep; frequent consumption of fast food, spicy food, coffee, and tea; BMI greater than 25 kg/m^2; and age did not show statistical significance with IBS.	The study observed a high prevalence of IBS in the Central Saudi Arabian population, accompanied by several modifiable risk factors. However, it's important to note that the sample size of this study was relatively small, suggesting a need for more extensive research.
Hafiz et al. 2023 [32]	In the Makkah region, 420 out of the 936 participants, or 44.9%, were identified as having IBS. Most of the IBS patients in this sample were women, aged between 25 to 35 years, married, and presented with mixed IBS.	Several risk factors were found to be associated with IBS, including age, gender, marital status, and occupation. In the age group distribution, the majority with IBS (48.3%) were between 25 and 35 years old. IBS was more prevalent in females (59.8%) than in males (40.2%). The study also found associations between IBS and various medical and lifestyle characteristics. These included insomnia, regular medication use, food allergies, chronic health issues, family history of IBS, history of gastroenteritis, and body mass index. In contrast, no significant association was found between IBS and factors such as history of pelvic surgery, smoking, caffeine intake, exercise, food source, education level, or nationality.	The researchers expressed hope that their findings would encourage further research and initiatives to enhance the quality of life for those affected by IBS.
Taha et al. (2019) [33]	The study found that the prevalence of Irritable Bowel Syndrome (IBS) among healthcare professionals in Al-Madinah City's primary healthcare centers was 16%. This amounted to 33 out of the 205 participants showing symptoms or a diagnosis of IBS.	The univariate analysis showed several factors associated with IBS, including nationality (with non-Saudis having a higher prevalence), anxiety, depression, performance, and dysphoria. Those with IBS had higher scores on the anxiety (11.4 ±3.2) and depression (11.7 ±3.4) scales compared to those without IBS (8.9 ± 2.7 for anxiety and 6.3 ± 2.4 for depression). Moreover, IBS participants scored higher in dysphoria (15.8 ±3.8) compared to non-IBS participants (11.3 ±3.1). In the multivariate analysis, the most significant predictor for IBS was found to be the depression score.	The multivariate analysis also indicated that the depression score was a significant predictor of IBS. The findings emphasize the substantial association between psychological distress, especially depression, and the presence of IBS among the participants.
Bashir Fadl et al. 2022 [34]	Irritable Bowel Syndrome (IBS) had a notably high prevalence among undergraduate medical students in Saudi Arabia, at 49.3%. This prevalence rate is higher than the global average, which typically falls between 5.7% and 34%.	Several factors were associated with the occurrence of IBS in the study population. Specifically, the study identified female gender, being in a higher academic year, sleeping less than 6 hours, and less frequent exercise as contributing factors to the likelihood of having IBS. Furthermore, there was a mention that more than half (56.7%) of the participants slept more than 6 hours and less than half (47.0%) practiced physical exercise regularly.	The study concluded with a recommendation for more proactive screening and management of stress factors, especially given the elevated prevalence of IBS among medical students.
Hasosah et al. 2017 [35]	The prevalence of IBS among the participants was 15.64%. Out of this, males had a higher diagnosed rate of 13% compared to females. The difference in IBS prevalence between males and females was not statistically significant (p > 0.05).	Several risk factors were associated with IBS. A high level of stress was significantly linked to IBS, with 7.26% of those with high stress diagnosed with the condition compared to 8.38% with low stress. Furthermore, having a family history of IBS and a lack of exercise were also significant risk factors for IBS, with p-values of 0.045 and 0.0229, respectively. The academic year level of the students, however, was not a risk factor for IBS.	IBS is a notable health concern among medical students. Factors like stress, a family history of IBS, and a lack of physical activity predispose individuals to the condition.
Alshammari et al. 2016 [36]	Out of the 207 subjects, aged between 20-60 years, the overall prevalence of IBS was found to be 35.7%.	Multivariate analysis revealed that the average family income per month had a significant impact on the occurrence of IBS (P<0.05). However, other factors such as age, marital status, sector of the workplace, and educational level did not significantly affect the prevalence of IBS among the studied group (P>0.05).	IBS is notably prevalent among educated and working women in Arar, KSA. Additionally, given the stressful nature of their work, stress management courses are suggested as a primary requirement to help these women cope and manage work-related stressors.
Aldawsari et al. 2017 [37]	The study revealed that 9.6% of the male medical students at Majmaah University claimed to have IBS, but only 7.3% were previously diagnosed with the condition.	The results indicated a higher prevalence of IBS among students with a GPA higher than 4. Specifically, 8 students (or 72% of those with IBS) had a high GPA, while only 3 students (or 27.3% of those with IBS) had a low GPA. However, this association was found to be not statistically significant (p=0.635). Among the diagnosed cases, the mixed subtype of IBS was the most common, accounting for 61% of the cases. The constipation and diarrhea subtypes constituted 22.1% and 16.9% respectively	. The study ultimately concluded that IBS prevalence among Majmaah University's medical students was on the lower side and that the mixed subtype was the most common form of the condition. Furthermore, a higher prevalence was observed among students with a GPA higher than 4.

Prevalence Rates

The prevalence rates for IBS among the studies varied, ranging from 7.9% to as high as 49.3% [18-37]. The average prevalence across these studies is approximately 24%.

High prevalence: Rates exceeding 40% were seen among school teachers in the Qassim region (40.7%) [18] and undergraduate medical students (49.3%) [34]. Moderate-to-high prevalence rates of around 30% were observed in the Central Saudi Arabia population (30.5%) [31] and educated and working women in Arar, KSA (35.7%) [36].

Moderate prevalence: Noteworthy were the findings from the south-west area of Saudi Arabia (23.81%) [27], healthcare professionals in Al-Madinah (16%) [33], physicians and surgeons across Saudi Arabia (16.3%) [29], and Makkah region's general population (44.9%) [32].

Low prevalence: Lower rates were documented in studies involving other male medical students at Majmaah University (9.6%) [37] and medical students in other regions (12.6%) [30].

Prevalence by Age and Occupation

While age brackets were not consistently detailed, some studies, such as the one by AlKhalifah et al. [18] and Hafiz et al. [32], highlighted the significant representation of the 31-40 age bracket. The vulnerability of specific populations such as students, teachers, and healthcare professionals are evident, with varying prevalence rates across the different studies.

Risk Factors

A trend suggests a higher IBS prevalence in females, though some studies reported slightly higher male prevalence. Regarding behavioral and health background, emotional stress, anxiety, and depression were recurrent risk factors. Notably, past smokers in the Makkah study [27] and current smokers in other studies, such as Ahmed et al. [30], showed a higher propensity for IBS.

Factors such as reduced water intake, fast food, spicy food, coffee, and tea were recurrent in some studies, though not all had statistical significance.Family history was a strong risk factor in various studies, with unique factors such as a history of gastroenteritis emerging in studies like the one from the Makkah region [32].

Medical students, healthcare professionals, and teachers showed notably varying prevalence rates. Of significance was the extremely high prevalence among undergraduate medical students (49.3%) [34] and school teachers in the Qassim region (40.7%) [18]. IBS prevalence in Saudi Arabia is diverse across studies and demographics. The variations arise from methodological differences, population differences, and potential biases. Factors such as gender, age, occupation, lifestyle, and mental health significantly influence IBS risk. Awareness, management, and mitigation remain pivotal for the health and wellbeing of the Saudi population.

Discussion

The prevalence and associated risk factors of IBS have been the focus of several studies in Saudi Arabia, spanning diverse populations ranging from school teachers to university students and general residents. The prevalence rates for IBS among the studies varied, ranging from 7.9% to as high as 49.3% [18-37]. The average prevalence across these studies is approximately 24%.

The review incorporates 17,018 participants, shedding light on their sociodemographic attributes and the research methodologies adopted. Notably, the range of sample sizes is vast, with a minimum of 115 participants [37] and a maximum reaching 6,300 [27]. Male participation fluctuates considerably across studies, with an average representation of about 63%. Geographically, the research casts a wide net over Saudi Arabia, and age specifics also varied, with some studies providing precise averages or ranges and others omitting age details altogether. When pooling data from those providing clear age averages, the aggregated mean age surfaced at roughly 28.15 years. Probably due to many studies being done on students in universities. We also believe that many studies being done on students also contributed to the high level of prevalence of IBS in this review; that is because of the high level of stress students face especially in academically demanding schools, such as medical and scientific faculties.

A comprehensive analysis of 53 research studies from 38 different countries using the Rome III criteria found an average prevalence of 9.2%, a figure closely resembling some of the studies mentioned observations. In contrast, a study conducted in Iran showed a substantially lower prevalence rate of 1.1%, based on the Rome III criteria [21,38,39]. One potential reason for these differing outcomes might be the application of Western diagnostic criteria for IBS in Eastern nations, which might not be fully aligned with the region's cultural and linguistic nuances. Other factors contributing to these discrepancies could include the adoption of varying criteria, smaller research sample sizes, and ethnic differences.

Starting with the school teacher cohort, AlKhalifah et al. (2016) recorded a notably high prevalence of IBS among teachers in the Qassim region at 40.7% [18]. Notably, only 35.2% of those teachers had previously been diagnosed with the condition. A gender-based breakdown showed a marginally higher prevalence in males than females. There is a slightly higher prevalence among the older age groups, specifically between 51 and 60 years, followed closely by those aged 31-40 years. Absenteeism, a factor linked to productivity and job performance, was significantly higher among teachers with IBS, pointing to potential ramifications on the education system.

Comparatively, studies among student populations showed varying results. Hakami et al. (2019) found a prevalence of 8.8% among university students at Jazan University [19]. This discrepancy in prevalence between university students and school teachers could be attributed to variations in the methodology, the population studied, or genuine differences in prevalence due to lifestyle or age-related factors. Factors such as educational discipline, living conditions, behaviors, and mental health showed substantial influence. Furthermore, Alharbi et al. (2022) identified a prevalence of 20.19% in the general population of Makkah Al-Mukarramah City [20]. The most common subtype of IBS was IBS-M at 53.8%. Psychiatric disorders, especially stress, were found to be significantly associated with IBS.

Amin et al. (2021) reported a 7.9% prevalence in the Saudi population, with females being more affected than males [21]. Socioeconomic factors, such as income and employment status, were highlighted, but food restrictions and knowledge about herbal remedies for pain relief were also addressed. The relationship between food restrictions and IBS hints at dietary factors playing a role in symptom management.

Further highlighting the variations in IBS prevalence across regions, Alqahtani et al. (2019) reported an 18.2% prevalence rate in the national survey [22]. Family history emerged as the most pronounced risk factor, with a whopping 80% of the patients reporting it. This suggests a possible genetic or shared environmental component to the disease.

This research highlighted a notably higher frequent use of NSAIDs among IBS patients. Similarly, Keszthelyi et al. in a case-control research identified that NSAIDs were used more often by those with IBS compared to the control group. It is theorized that NSAIDs may amplify intestinal permeability in IBS patients, permitting luminal antigens to access the lamina propria, which in turn triggers an immune and inflammatory response.However, more in-depth prospective cohort studies are required to validate the role of NSAIDs as a potential risk factor for IBS and to better understand the underlying cause of this link [22,40,41].

Dietary habits, especially low water intake and lack of dietary fiber from fruits and vegetables, were significantly associated with IBS in the study by Alharbi et al. [23]. Saad Alshahrani found that IBS symptoms were present in 39.8% of male secondary school students in Najran City [24]. Notably, a positive family history and the presence of diabetes mellitus were highlighted as significant risk factors.

While the included study did not definitively link caffeine to IBS, there was a marginal rise in risk associated with its use. Caffeine, a widely consumed psychoactive compound globally, is valued for its ability to stimulate the central nervous system. Beyond traditional sources such as coffee, tea, and sodas, some individuals are now gravitating toward the latest trend, energy drinks, for their caffeine fix. Predominantly, coffee is known to boost gastric acid production and influence colonic motor activity. The National Institute for Health and Care Excellence (NICE) recommends that daily intake of coffee and tea should not exceed three cups [23,41-43]. Other studies, such as Alnasser et al., emphasize the impact of IBS on the health-related quality of life (HRQoL), especially among elderly women [25]. Lastly, Ibrahim et al. showcased a prevalence of 33.3% among paramedical students at King Abdulaziz University, Jeddah [26]. Gender and educational specialty were vital factors, with nursing and dentistry students showing the highest prevalence.

Sachithananthan [28] indicated a very high prevalence of 53.3% among participants, highlighting the influence of factors such as psychological stress or anger on the onset of IBS symptoms.

The prevalence of IBS among professionals in the medical field was of particular note. AlAmeel's 2019 study [29] revealed a 16.3% prevalence among surveyed physicians and surgeons, with significant variance depending on specialties. A key insight from this study was the role of age, gender, and work hours in predicting IBS occurrence. This sentiment echoed among medical students. Ahmed et al. [30] found a 12.6% prevalence among their sample of medical students, suggesting a potential link between smoking and IBS prevalence.

The intricate relationship between IBS and mental health is evident in various studies. Aljammaz et al. [31] found that, out of their participants, 13.8% had depression and 28.2% had anxiety, both of which were significantly associated with IBS. This link between psychological distress and IBS is further substantiated by Taha et al. [33], wherein they found higher scores of anxiety and depression among those diagnosed with IBS.

Other studies expanded the demographic focus. In the Makkah region, Hafiz et al. [32] reported a prevalence of 44.9% for IBS. Their study further elucidated risk factors, including age, gender, marital status, and occupation. Bashir Fadl et al. [34] discovered a strikingly high prevalence of 49.3% among undergraduate medical students, a rate higher than most global averages. This particular study emphasized the potential influence of stress factors, especially given the academic demands.

Societal and lifestyle factors further contribute to this health concern. Hasosah et al. [35] found stress, a family history of IBS, and a lack of physical activity as significant predictors of IBS. Alshammari et al. [36] identified a notable prevalence of 35.7% in their sample, indicating potential associations with family income levels. Lastly, Aldawsari et al. [37] reported a relatively lower prevalence of 9.6% among male medical students at Majmaah University, underscoring the possibility that academic performance, indicated by GPA, could be linked to IBS, albeit not significantly.

Risk factors for IBS can be broadly categorized into several domains: genetic, environmental, physiological, and psychological. The studies cited from Saudi Arabia provide insights into how these domains intersect and are reflected in the Saudi population. Several studies highlighted the significance of family history as a risk factor [22,24]. Alqahtani et al. revealed that 80% of IBS patients reported a family history of the condition, suggesting a potential genetic predisposition or shared environmental influences within families [22]. While the exact genes responsible for IBS are not yet identified, the pronounced family association warrants further genetic research to elucidate any hereditary markers or susceptibility genes.

Gender differences are evident in the prevalence of IBS. In some studies, females were more affected than males [21]. Hormonal changes, especially in women, are believed to influence gut function and sensitivity, possibly explaining the gender disparity. Age is another factor, with some age groups, such as the 51-60-year bracket, showing a higher prevalence [18]. The reasons could be multifactorial, ranging from accumulated life stressors, changes in dietary habits, or physiological alterations that come with aging.

Diet plays a pivotal role in IBS, both as a trigger and a potential management tool. A study by Alharbi et al. highlighted the association of low water intake and insufficient dietary fiber from fruits and vegetables with IBS [23]. The gut's microbial environment is significantly influenced by diet, and imbalances in this environment might contribute to IBS symptoms. Foods high in FODMAPs (fermentable oligo-, di-, mono-saccharides, and polyols) have been recognized globally as potential triggers for some IBS patients, reinforcing the dietary connection.

Stress and psychiatric disorders, particularly anxiety and depression, are consistently associated with IBS [20]. The gut-brain axis, a bidirectional communication system between the gastrointestinal tract and the central nervous system, plays a central role in this association. Emotional stressors can lead to physiological responses in the gut, exacerbating IBS symptoms. Conversely, chronic gastrointestinal symptoms can induce or aggravate stress and anxiety, creating a feedback loop.

Certain medical conditions, such as diabetes mellitus, were identified as significant risk factors in some cohorts, such as the male secondary school students in Najran City [24]. The interplay between IBS and other diseases can be complex. For instance, diabetes can cause autonomic neuropathy affecting gut motility, potentially leading to IBS-like symptoms.

Factors such as educational discipline, living conditions, and socioeconomic status were found to influence IBS prevalence in some studies [19,21]. For instance, university students from specific disciplines, such as nursing and dentistry, exhibited a higher prevalence of IBS [26]. This suggests that occupational stress or lifestyle habits related to specific disciplines might contribute to IBS development or exacerbation.

In summary, IBS is a multifactorial disorder, with numerous risk factors associated with onset and progression. Recognizing and understanding these factors is crucial for early diagnosis. The pronounced impact of some of these risk factors, as reflected in the Saudi population, emphasizes the need for localized research and interventions tailored to the region's unique sociocultural and genetic makeup.

Strengths and Limitations

The following are some strengths of this thorough literature review: In order to avoid reviewer biases as much as possible, we feel that (a) we followed PRISMA recommendations and used reputable methods for data extraction and quality evaluation, and (b) we presented all study findings graphically to the reader. As for limitations, the study included only freely accessible articles on the topic. Additionally, the heterogeneity of the studies prevented us from performing a meta-analysis. The ability of analyses to attain significance may be constrained by the small sample sizes and methodological problems in some of the included studies. The validity and generalizability of the results in some studies with small sample numbers and high rates of loss-to-follow-up may be compromised by selection bias.

## Conclusions

In conclusion, the highlighted studies provide a comprehensive overview of the IBS landscape in Saudi Arabia. The variations in prevalence across different populations and regions suggest multifaceted determinants, including genetic, environmental, lifestyle, and possibly occupational. Gender differences, age groups, family history, and dietary habits all seem to influence IBS prevalence. The consistent recognition of the condition's impact on the quality of life underscores the necessity for heightened awareness, early diagnosis, effective management, and possibly intervention programs tailored to the specific needs and risk factors of various demographic groups.
